# Soybean kinome: functional classification and gene expression patterns

**DOI:** 10.1093/jxb/eru537

**Published:** 2015-01-22

**Authors:** Jinyi Liu, Nana Chen, Joshua N. Grant, Zong-Ming (Max) Cheng, C. Neal Stewart, Tarek Hewezi

**Affiliations:** Department of Plant Sciences, University of Tennessee, Knoxville, TN, USA

**Keywords:** abiotic stress, biotic stress, co-expression network, collinearity analysis, duplication events, gene expression, soybean kinases.

## Abstract

The protein kinase (PK) gene family is one of the largest and most highly conserved gene families in plants and plays a role in nearly all biological functions. While a large number of genes have been predicted to encode PKs in soybean, a comprehensive functional classification and global analysis of expression patterns of this large gene family is lacking. In this study, we identified the entire soybean PK repertoire or kinome, which comprised 2166 putative PK genes, representing 4.67% of all soybean protein-coding genes. The soybean kinome was classified into 19 groups, 81 families, and 122 subfamilies. The receptor-like kinase (RLK) group was remarkably large, containing 1418 genes. Collinearity analysis indicated that whole-genome segmental duplication events may have played a key role in the expansion of the soybean kinome, whereas tandem duplications might have contributed to the expansion of specific subfamilies. Gene structure, subcellular localization prediction, and gene expression patterns indicated extensive functional divergence of PK subfamilies. Global gene expression analysis of soybean PK subfamilies revealed tissue- and stress-specific expression patterns, implying regulatory functions over a wide range of developmental and physiological processes. In addition, tissue and stress co-expression network analysis uncovered specific subfamilies with narrow or wide interconnected relationships, indicative of their association with particular or broad signalling pathways, respectively. Taken together, our analyses provide a foundation for further functional studies to reveal the biological and molecular functions of PKs in soybean.

## Highlight

Identification and functional classification of soybean kinase gene family revealed wide expansion and extensive divergence in gene structure, subcellular localizations, and tissue and stress gene expression patterns.

## Introduction

The protein kinase (PK) gene family is one of the largest and most highly conserved gene families in plants. PKs phosphorylate proteins for functional changes and are involved in nearly all cellular processes, thereby regulating almost all aspects of plant growth, development, and responses to biotic and abiotic stresses. PK genes exist by the hundreds in all plant species in which they have been surveyed. Genome-wide identification of PKs in a number of plant species have indicated the presence of more than 3% of the annotated proteins coding for PKs, an indication of their functional importance (Lehti-Shiu *et al.*, 2009; Lehti-Shiu and Shiu, 2012). The functional classification of PKs was initially conducted based on the conservation and phylogeny analysis of the catalytic domains of eukaryotic PKs (Hanks *et al.*, 1988; Hanks and Hunter, 1995). These classifications resulted in defining five major groups, divided into 55 subfamilies, with related substrate specificity and mode of regulation. The Hanks and Hunter classification was extended further by Manning *et al.* (2002) and Niedner *et al.* (2006) where the entire PK superfamily was classified into nine groups, 81 families, and 238 subfamilies based on sequence comparison of the catalytic domains as well as sequence similarity and domain structure outside the catalytic domains. Very recently, PKs from 25 plant species were classified into nine main groups and 115 families (Lehti-Shiu and Shiu, 2012). The defined groups included AGC (PKA–PKG–PKC), CAMKs (calcium- and calmodulin-regulated kinases), CK1 (casein kinase 1), CMGC (including *c*yclin-dependent kinases, *m*itogen-activated protein kinases, *g*lycogen synthase kinase and *c*yclin-dependent kinases), STE (including many kinases functioning in MAP kinase cascades), TK (tyrosine kinases), TKL (tyrosine kinase-like kinases), plant-specific, and ‘other’ (a group of kinases that could not be classified easily into the previous groups). Each plant PK repertoire or kinome is significantly larger in size than that of other eukaryotes, including those residents in animal genomes. The dramatic expansion of plant kinomes over other eukaryotes may be the result of recent duplication events and a high retention rate of duplicates (Hanada *et al.*, 2008; Lehti-Shiu and Shiu, 2012).

Several studies have indicated that extensive expansion of specific PK families such as receptor-like kinase (RLK)/Pelle has also contributed to the large number of plant PKs (Shiu and Bleecker, 2003; Shiu *et al.*, 2004; Vij *et al.*, 2008; Lehti-Shiu *et al.*, 2009; Gao and Xue, 2012; Wei *et al.*, 2014). The RLK/Pelle family has expanded at a significantly higher rate than other kinases and in general constitutes more than 60% of the PKs in flowering plants. The large size of this family can be attributed to the expansion of a few subfamilies, specifically those bearing leucine-rich repeat (LRR) domains (Lehti-Shiu *et al.*, 2009). Because members of these subfamilies are known to have roles in defence/resistance responses, it has been suggested that the wide expansion was probably a result of adaptation to rapidly evolving pathogens (Lehti-Shiu *et al.*, 2009). In contrast, the RLK subfamilies with developmental functions are conserved in size (Shiu *et al.*, 2004). The expansion of the PK superfamily seems to correlate well with increasing developmental complexity. For example, the green alga *Ostreococcus tauri* and moss (*Physcomitrella patens*) contain 93 and 685 PKs, respectively, compared with 1008 PKs in *Arabidopsis*. In addition, there are considerable differences in the sizes of kinomes among plant species by more than 4-fold. However, because the kinome of various plant species constitutes a relatively similar percentage of the whole-genome protein-coding genes, it seems likely that the PK superfamily has undergone similar mechanisms of expansion in flowering plants (Lehti-Shiu *et al.*, 2009).

Functional characterization studies of PKs in *Arabidopsis thaliana* during the last two decades have positioned PKs as core components of various signalling pathways controlling multiple biological processes (Champion *et al.*, 2004; Morillo and Tax, 2006; Colcombet and Hirt, 2008; Rodriguez *et al.*, 2010; Gish and Clark, 2011; Tena *et al.*, 2011). In soybean, however, only a limited number of PKs have been functionally characterized, despite the recent development of functional genomics tools and a published soybean genome (Schmutz *et al.*, 2010). Soybean PKs have been found to function in the response to biotic stress (Srour *et al.*, 2012) and abiotic stress (Im *et al.*, 2012; Sun *et al.*, 2013; Yang *et al.*, 2014), as well as in the equilibrium of gene expression between disease resistance and growth and development (Liu *et al*., 2011, 2014).

Whole-genome sequencing of several plant species has allowed large-scale identification of plant kinomes with higher resolution than earlier studies. With the availability of the soybean whole-genome sequences along with annotation of the encoded proteins (Schmutz *et al.*, 2010), it is now possible to identify and functionally classify the PK family using large-scale phylogenetic approaches, which was our goal for this study. We identified 2166 putative PK genes, which were functionally classified into groups, families, and subfamilies. Our analysis of soybean PKs also included their chromosomal location, gene structure, duplication events, expansion, subcellular localization, expression profiles, and co-expression relationships.

## Materials and methods

### Identification and classification of soybean PKs

To identify soybean PKs at the genome level, all protein-coding genes were downloaded from the latest version of the soybean genome (Wm82.a2.v1) from Phytozome (v. 10.0.). Hidden Markov models (HMMs) of the ‘typical’ Pkinase clan [Pkinase (PF00069) and Pkinase_Tyr (PF07714)] (Finn *et al.*, 2010) were used to search for putative PKs using HMMER v. 3.0 (Finn *et al.*, 2011) with an E-value cut-off of <1.0. After this initial screen, 2352 sequences containing the PK domain were identified. Only the longest variant of each gene was retained and all other redundant sequences were deleted. All the remaining sequences were further aligned with the PFAM kinase domain models to confirm the presence of kinase domains (Pkinase and Pkinase_Tyr domains) and eliminate pseudogenes as described previously by Lehti-Shiu and Shiu (2012). In this analysis, the putative PKs were considered typical PKs only if the domain alignments covered at least 50% of the PFAM domain models. Finally, a total of 2166 proteins containing at least one kinase domain were identified and assigned as soybean ‘typical’ PKs. Classification of identified kinases to groups, families, and subfamilies was defined using HMMs of the different subfamilies developed by Lehti-Shiu and Shiu (2012) based on PK sequences obtained from 21 plant species. The classification was further confirmed using phylogenetic approaches using neighbour-joining (NJ) and maximum-likelihood (ML) methods.

### Chromosome location and synteny analysis of soybean PKs

Information about the chromosome locations of all soybean PKs was obtained from the Phytozome database. Putative homologous chromosomal regions and segmental/tandem duplication events of the PK superfamily were determined using the Multiple Collinearity Scan toolkit (MCScanX, http://chibba.pgml.uga.edu/mcscan2/) (Wang *et al*., 2012c, 2014). Synonymous (*K*_s_) and non-synonymous substitution (*K*_a_) rates were calculated using the ‘add_ka_and_ks_to_collinearity.pl’ function of MCScanX. Collinearities of duplicated PK genes were visualized using the Genome Pixelizer program (http://www.atgc.org/GenomePixelizer). Tandemly repeated PKs in soybean genome were mapped to position on each chromosome with Mapchart v2.2 (https://www.wageningenur.nl/en/show/Mapchart.htm).

### Intron number

Intron numbers of all PKs were obtained by surveying the GFF file from Phytozome v. 10.0 (http://phytozome.jgi.doe.gov/pz/portal.html#!info?alias=Org_Gmax) using Perl script.

### Subcellular localization

To determine the putative transmembrane proteins of soybean PKs, the N-terminal signal peptides and transmembrane domains were predicted using the SignalP v. 4.1 (Petersen *et al.*, 2011) and TMHMM v. 2.0 (Krogh *et al.*, 2001) programs, respectively. The subcellular localization of soybean PKs was predicted by WoLF PSORT (Horton *et al.*, 2007). To test the predicted nuclear localization patterns *inplanta*, the coding sequences of six soybean PKs (Glyma.02G276000, Glyma.04G029700, Glyma.14G000800, Glyma.04G097100, Glyma.11G050000, and Glyma.12G182600) were amplified using gene-specific primer pairs containing *Xho*I and *Sal*I restriction enzyme sites in the forward and reverse primers, respectively. PCR amplification was performed using the high-fidelity PrimeSTAR GXL DNA polymerase (Clontech) according to the manufacturer’s instructions. PCR products were digested by *Xho*I and *Sal*I, gel purified, and fused to the N terminus of the yellow fluorescent protein (YFP) reporter gene in the pSAT6-EYFP-N1 vector and under the control of a double 35S promoter. The constructs were delivered into onion epidermal cells by biolistic bombardment as described previously by Hewezi *et al.* (2010). After bombardment, onion epidermal peels were incubated at 26 °C for 24h in the dark. The subcellular localization of the fused proteins was visualized using an EVOS^®^ FL Auto Cell Imaging System (Life Technologies). The subcellular localization of each protein was determined at least three times.

### Microarray data analysis

To analyse the expression patterns of soybean PKs for tissues and stress treatments, microarray data were retrieved from the Gene Expression Omnibus (GEO) database at the National Center for Biotechnology Information. Fifty microarray datasets for various tissues and organs and 24 datasets for biotic and abiotic stress treatments were selected from the largest Affymetrix platform, GPL4592. The raw data were normalized using the Affymetrix MAS5 algorithm using the R package Affy (Gautier *et al.*, 2004). The normalized gene expression values of 817 individual PK genes were used to calculate the expression value for each gene relative to the mean expression level of each tissue/organ included, as described previously (Schmid *et al.*, 2005) or relative to the corresponding controls of stress treatments. In the case of subfamily, the normalized gene expression values were averaged for all members within each subfamily and used to calculate the expression value for each subfamily as described above. Because only 817 out of 2166 PKs have probes on the Affymetrix microarray GPL4592 platform, we included in our analysis only subfamilies in which gene expression data were available for at least one-third of the subfamily members. Heat maps of expression levels of individual PK genes (817 genes) or 82 PK subfamilies were generated using MeV (MultiExperiment Viewer) software, v. 4.9 (http://www.tm4.org/mev.html).

### Co-expression networks

Co-expression networks were generated for 82 PK subfamilies where gene expression data were available for at least one-third of the subfamily members. For each tissue and stress treatment, gene expression values for all members in each subfamily were averaged and the Pearson correlation coefficient (PCC) was computed between each pair of the subfamilies using SPSS v. 20.0. Co-expressed subfamilies were selected with a PCC cut-off of 0.7. This cut-off value was used previously in several studies and is well suited to generate moderate-sized networks (Lin *et al.*, 2011; Wang *et al.*, 2012*b*; Lan *et al.*, 2013). Two independent co-expression networks were generated using tissue and stress response gene expression data described above. The constructed co-expression networks were visualized using the Cytoscape v. 3.1.1 program (http://www.cytoscape.org/) (Saito *et al.*, 2012).

## Results

### Genome-wide identification and classification of soybean PKs

**Fig. 1.**
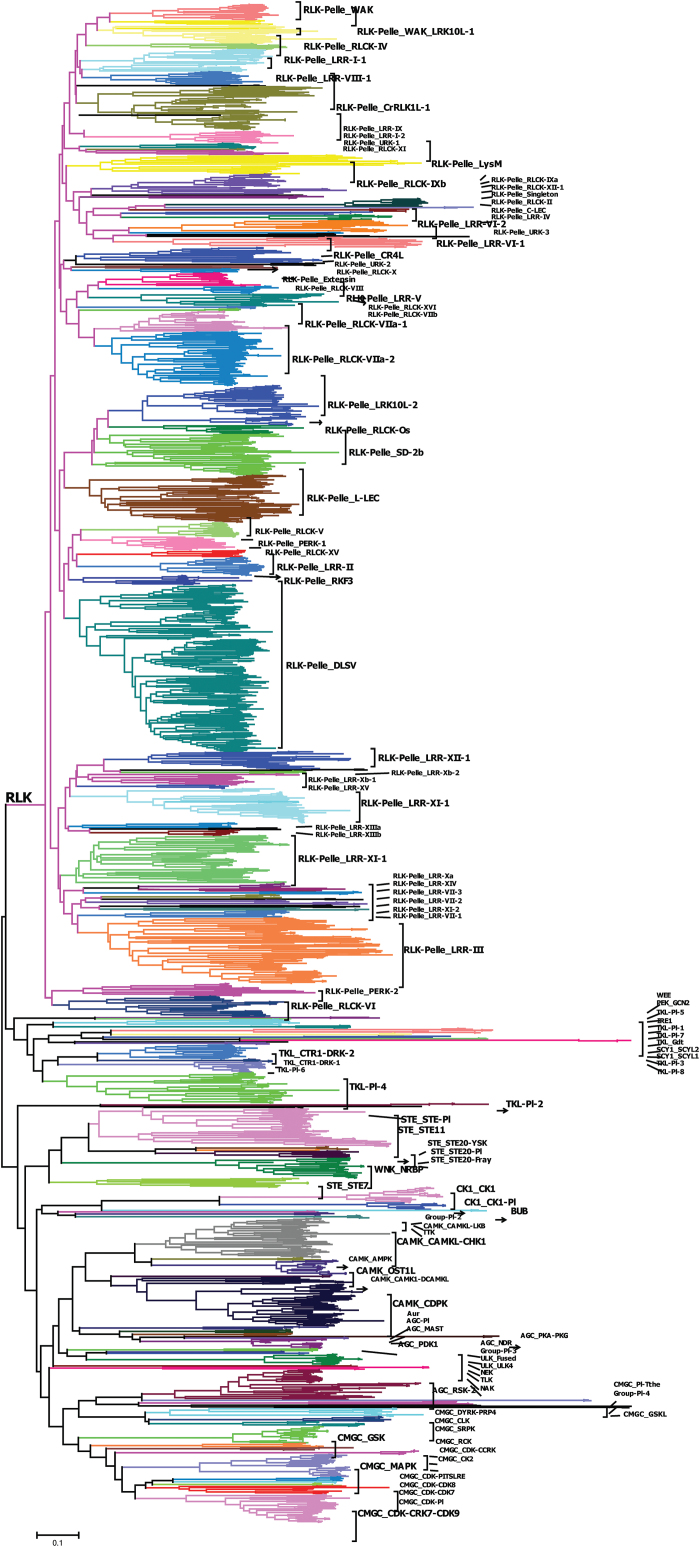
Classification and phylogenetic relationships of soybean PK subfamilies. The phylogenetic tree was constructed using MEGA6 software, with the NJ method using kinase domain amino acid sequences. Subfamilies are highlighted with different colours. The RLK group basic branch is labelled. Detailed information of the phylogeny and the corresponding HMM classification are provided in Supplementary Fig. S1 and Supplementary Table S3. (This figure is available in colour at *JXB* online.)

We searched all annotated genes of the latest version of the soybean genome (Wm82.a2.v1) for genes encoding PKs using HMMER v. 3.0 (Finn *et al.*, 2011) with HMMs of the ‘typical’ Pkinase clan (Pkinase and Pkinase_Tyr) (Finn *et al.*, 2010). When the putative PK sequences were aligned with PFAM kinase domain models, we down selected genes for further analysis if the domain alignments covered at least 50% of the PFAM domain models, which resulted in the identification of 2166 PKs (Supplementary Table S1 at *JXB* online) and exclusion of 186 atypical kinases (Supplementary Table S2 at *JXB* online). The soybean kinome was further classified into groups and subfamilies using the HMM search approach described in Materials and methods and confirmed by phylogenetic tree analysis using NJ (Fig. 1 and Supplementary Fig. S1 at *JXB* online) and ML methods (Supplementary Fig. S2 at *JXB* online). Interestingly, both NJ and ML methods generated the same classification for 2152 PKs, and only 14 genes showed different classification between both methods including 12 unclassified genes by the NJ method (Supplementary Table S3 at *JXB* online). The 2166 soybean PKs were then classified into 19 groups in which RLK (1418), CMGC (166), CAMK (158), TKL (115), STE (86), AGC (70), and CK1 (32) comprised 2045 genes, representing 94% of the soybean kinome. These 19 groups were further classified into 122 subfamilies (Supplementary Table S3). The size of the subfamilies was highly variable and varied between one gene (PEK_GCN2, TKL-Pl-3, and CMGC_GSKL) and 238 genes (RLK-Pelle_DLSV). Finally, a set of 20 PKs was arbitrarily classified by HMM search with low E-values but did not cluster with any of the known subfamilies and was thus assigned as an unclassified group (Supplementary Table S3). These genes might be novel and soybean specific, and might possibly constitute new gene families.

### Conserved domain and gene structure of soybean PKs

All the 2166 identified PKs were scanned against the PFAM database using HMMs to identify additional conserved domains that may be common among subfamilies. All identified conserved domains are provided in Supplementary Table S4 at *JXB* online. We found that 943 genes (43.5%) had only a kinase domain, whereas the remaining 1223 genes (56.5%) had additional conserved domains. A large fraction of PK genes with additional conserved domains was identified in the AGC (88.6%), RLK-Pelle (67.4%), CAMK (65.2%), and TKL (53%) groups (Supplementary Table S4), demonstrating that various subfamilies have various domain compositions. In contrast, members of each subfamily shared a common domain arrangement, suggesting common evolutionary history among PKs within the same subfamily. Interestingly, we found that 74 PKs contained two or three PK domains (Supplementary Table S5 at *JXB* online). These genes were found to be distributed among different subfamilies, but a significant proportion of these genes were found in specific subfamilies including RLK-Pelle_RLCK-XI (7/7), Group-Pl-2 (2/2), AGC_RSK-2 (38/43), CMGC_CDK-CCRK (3/4), and CMGC_SRPK (3/7) (Supplementary Table S5).

To gain further insights into the structural diversity of soybean PKs, we analysed the intron number of all soybean kinase genes. The number of predicted intronless genes (262) was found to be much less than that of intron-containing genes (1904) (Supplementary Table S3). The number of introns per gene varied from 0 to 28, with 336 (15.5%) genes containing more than 10 introns. Comparison of the exon/intron arrangement at the subfamily level revealed that the number of introns in 33 subfamilies was conserved in all subfamily members (Supplementary Table S3). The gene structure of some subfamilies was highly variable. For example, four members of the AGC_RSK-2 subfamily were intronless and 34 members contained one or two introns, whereas the five remaining members contained 21 introns. When we carefully examined the phylogenetic relationships of these genes, we found that members of this subfamily could be separated into two subclades. This analysis suggested that PKs with varied intron numbers within subfamilies had their own evolutionary trajectories during the course of evolution, which can be used as an evolutionary imprint for further phylogenetic classification of soybean PK subfamilies. While the majority of the soybean PK subfamilies included members with varied intron numbers, specifically those encompassing relatively many members, other families showed low numbers or intronless structures. For instance, the RLK-Pelle_CR4L family (23 members) contained 18 intronless genes and four genes with a one-intron/two-exon structure. Similarly, out of the 94 genes belonging to the RLK-Pelle_LRR-III subfamily, 59 had a single-intron structure. The intron/exon distribution patterns appeared to correspond well with the phylogenetic analysis and subfamilies classification, which provided another layer of verification of our classification process. Furthermore, we investigated the structural configurations of soybean PKs relative to other species, two eudicot species (*A. thaliana* and *Medicago truncatula*) and two monocot species (*Oryza sativa* and *Zea mays*), by comparing the number of introns across all PK genes. Interestingly, the distribution patterns of intron numbers in these five species were very comparable (Supplementary Fig. S3 at *JXB* online), suggesting that similar intron gain and loss events contributed to the structural evolution of the PK gene family before the eudicot–monocot split.

### Comparative analysis of the soybean kinase subfamilies with monocot and eudicot species

**Fig. 2.**
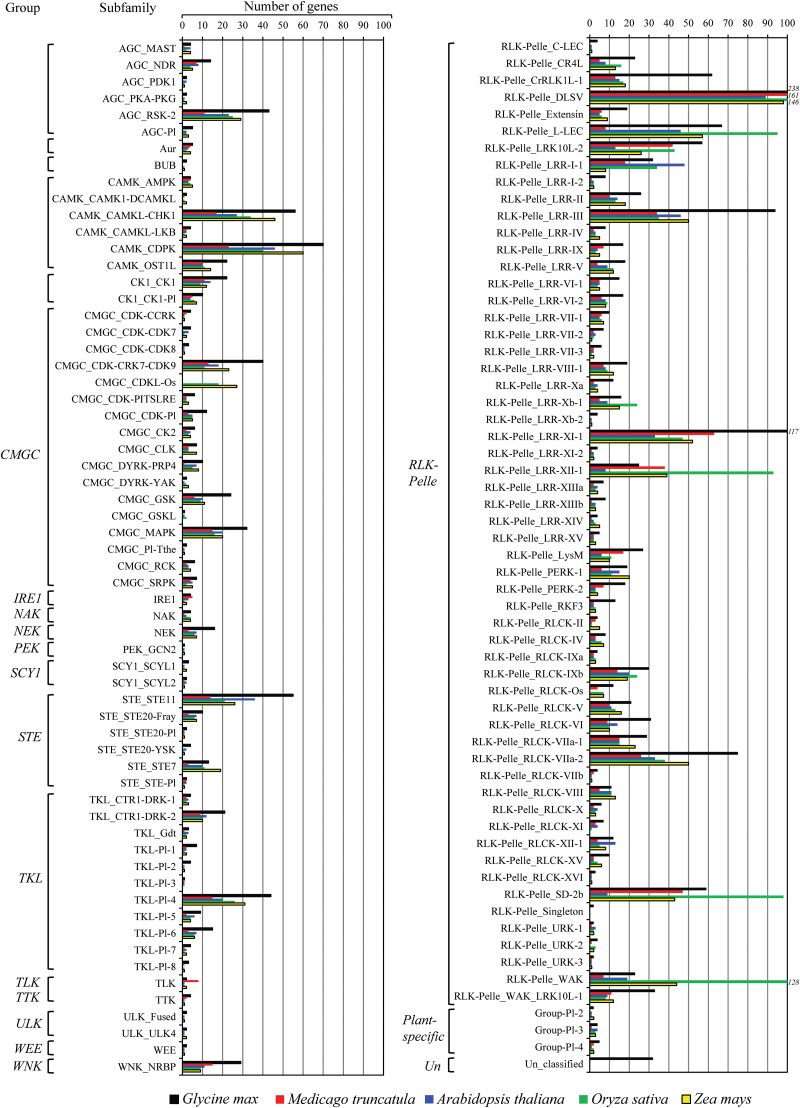
Comparison of the size of soybean PK subfamilies with other angiosperm species. The size of 122 soybean PK subfamilies was compared with those of four other plant species comprising two eudicot (*A. thaliana* and *M. truncatula*) and two monocot species (*O. sativa* and *Z. mays*). (This figure is available in colour at *JXB* online.)

We compared the distribution of soybean kinases across the 122 families with those of four other plant species comprising two eudicot (*A. thaliana* and *M. truncatula*) and two monocot (*O. sativa* and *Z. mays*) species. As expected, we found that the size of the majority of PK families was larger in soybean than in the other species, apparently because of its large kinome repertoire. Nevertheless, the expansion of the size of specific subfamilies belonging to the RLK-Pelle group was remarkable. For example, the RLK-Pelle_CrRLK1L-1 subfamily was comprised of 62 members in soybean, whereas the other four species contained between 13 and 18 members (Fig. 2). Other gene subfamilies including RLK-Pelle_DLSV, RLK-Pelle_LRR-III, RLK-Pelle_LRR-XI-1, RLK-Pelle_RLCK-VI, and RLK-Pelle_WAK_LRK10L-1 also contained much higher member numbers in soybean than in *Arabidopsis*, *M. truncatula*, rice, or maize. In addition, other non-RLK-Pelle subfamilies including AGC_RSK-2, CAMK_CAMKL-CHK1, TKL_Pl-4, CAMK_CDPK, and STE_STE11 contained much higher numbers of genes relative to the other four species. Thus, the large size of the soybean kinome could be attributed in part to the expansion of these specific subfamilies. In contrast, we found that 20 subfamilies contained only one or two members including, for example, PEK_GCN2, AGC_PDK1, AGC_PKA-PKG, CAMK_CAMK1-DCAMKL, CMGC_Pl-The, SCY1_SCYL2, STE_STE20-Pl, STE_STE-Pl, ULK_Fused, ULK_ULK4, RLK-Pelle_URK-1, and RLK-Pelle_URK-3 (Fig. 2). Notably, the small size of these subfamilies was also conserved in the other four plant species. It seems likely that these subfamilies have been subjected to limited expansion processes.

Two monocot-specific kinase subfamilies (CMGC_CDKL-Os and Others_BUB) were absent not only in soybean but also in the other two eudicot species (*A. thaliana* and *M. truncatula*) (Fig. 2). The CMGC_CDKL-Os kinase family is specific to monocots, and rice and maize contain, respectively, 18 and 27 genes belonging to this subfamily. Other subfamilies including CMGC_DYRK-YAK, CMGC_GSKL, PEK_PEK, plant-specific_Group-Pl-2, TKL_Pl-3, and RLK-Pelle_RLCK-XIII were identified in *Arabidopsis* and/or *M. truncatula* but were not detected in soybean, suggesting that these families have limited expansion ability and were subsequently lost in soybean. Consistent with this hypothesis, these six families are single-member gene families in *Arabidopsis* and *M. truncatula.*

### Chromosomal locations and segmental duplication events of PKs

**Fig. 3.**
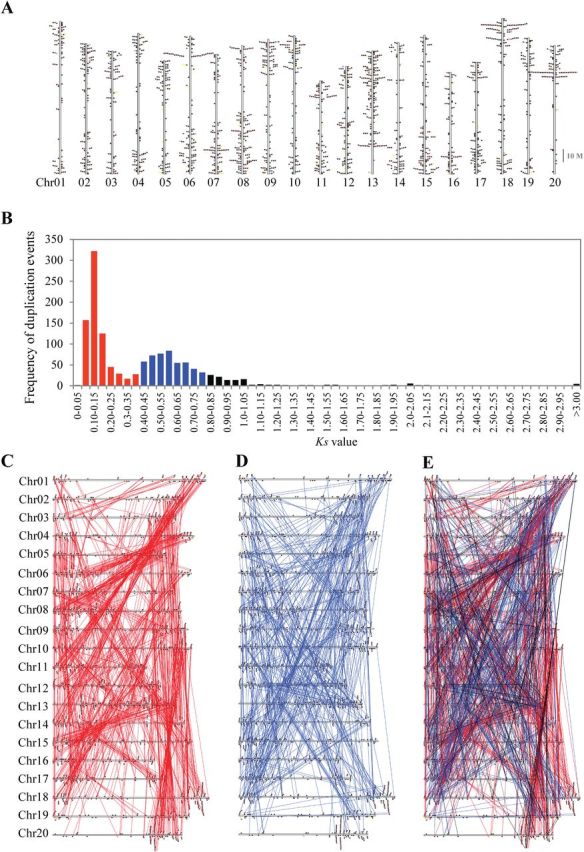
Chromosome location and collinearity of soybean PK genes. (A) Chromosomal locations of soybean PKs. The coloured boxes denote different groups of the soybean PK family. (B) Collinearity events among all duplicated PKs in the soybean genome. The bars denote collinearity events contributed by 13-Mya whole-genome-wide duplication (WGD) (*K*_s_ values 0.06–0.39) and by 59-Mya WGD (*K*_s_ values 0.40–0.80) and by other WGD events. (C, D) The collinearity events contributed by the 13-Mya (C) and 59-Mya whole-genome-wide duplication (WGD) events (D). (E) Contribution of the 13 and 59 Mya duplication events to the expansion of soybean PKs. The *K*_s_ values (synonymous distance) of collinearity events (1357) for all syntenically duplicated PKs (1547 genes) were calculated the MCScanX program. The *K*_s_ values of 0.06–0.39 were used to differentiate the events contributed by the 13-Mya WGD from those contributed by the 59-Mya WGD events (*K*_s_ values 0.40– 0.80). (This figure is available in colour at *JXB* online.)

All 2166 PKs were mapped to the 20 soybean chromosomes to define their genomic distribution and localization. The soybean PKs were localized mainly near the ends of the chromosomes (subtelomeric regions) and to a lesser extent in the non-telomeric and centromeric regions (Fig. 3A). The soybean PKs are not equally distributed across the 20 chromosomes. Chromosomes 8 and 13 contained the highest numbers of PKs per chromosome, 162 and 173 genes, respectively. In contrast, chromosomes 16 and 19 contained the lowest numbers of PKs per chromosome, with 78 and 74 genes, respectively. PK gene subfamilies were generally distributed on several chromosomes, with the exception of the RLK-Pelle_RLCK-Os gene family, which was located mainly on chromosomes 9 and 15 (Supplementary Table S3).

It has been suggested that the soybean genome experienced whole-genome-wide duplications (WGDs) approximately 59 and 13 million years ago (Mya) (Schmutz *et al.*, 2010). Therefore, we investigated the contribution of various duplication events to the expansion of soybean PKs. We first searched for segmental duplicates by examining soybean genome for duplicate blocks that contains kinase genes. We identified 1357 collinearity events among 1547 PK genes, indicating that segmental duplication events have played a major role in increasing the number of soybean PK genes (Supplementary Table S6 at *JXB* online).

Genome duplications are believed to have a significant impact on the expansion of certain gene families in plants, specifically those with regulatory functions (Maere *et al.*, 2005; Freeling, 2009). Thus, the contribution of the 13- and 59-Mya duplication events to the expansion of specific subfamilies was examined by determining the synonymous distance (*K*_s_ values) of collinearity events (1357) for all syntenically duplicated PKs identified above (1547 genes) (Fig. 3B). The *K*_s_ value of 0.06–0.39 was then used to differentiate the events contributed by the 13-Mya WGD from those contributed by the 59-Mya WGD events (*K*_s_=0.40–0.80) (Fig. 3B). We found 708 and 476 collinearity events arranged at the 13-Mya and 59-Mya WGD regions, respectively. The finding that both 13- and 59-Mya collinearity events were associated with 1470 PK genes out of 2166 (67.87%) indicated that the expansion of the soybean PK superfamily was significantly contributed by the 13- and 59-Mya WGD. Careful examination of the collinearity events of the 13- and 59-Mya WGD revealed their occurrence in several chromosomes at specific regions (Fig. 3C–E), suggesting that the expansion of soybean PKs was associated with polyploidization and subsequent genome shuffling.

Furthermore, we compared the collinearity events contributed by 13-Mya WGD (*K*_s_=0.06–0.39) and those contributed by the 59-Mya WGD (*K*_s_=0.40–0.80), to test the potential contribution of these two duplication events to the expansion of specific PK subfamilies. While both 13- and 59-Mya WGDs seemed to be responsible for the collinearity relationships of the majority of PK family paralogues, we identified 31 PK subfamilies in which collinearity events could be attributed only to the 13-Mya WGD (Supplementary Table S7 at *JXB* online). These 31 gene families may represent the evolution of additional PKs that function in specific developmental, environmental, or physiological conditions.

### Tandem duplication of soybean PKs

**Fig. 4.**
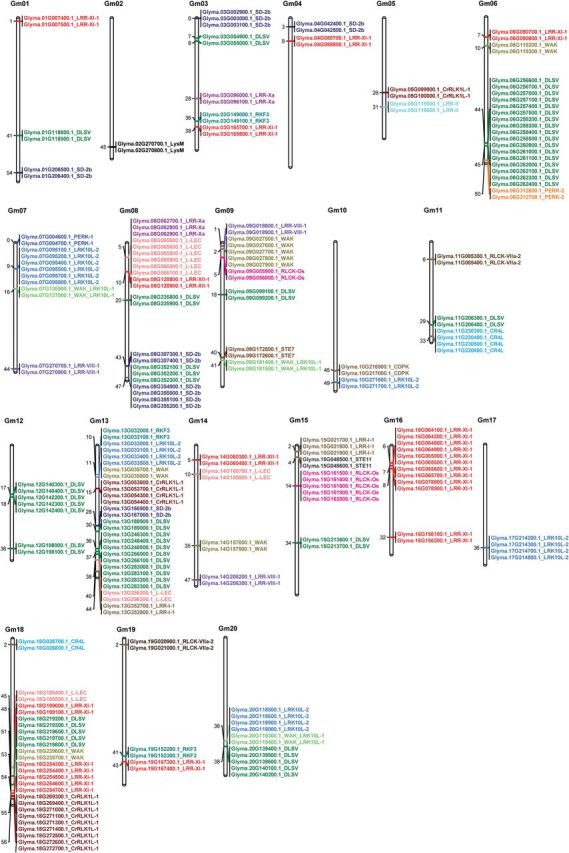
Chromosomal locations of the 229 tandemly arrayed soybean PK genes. The 229 tandemly arranged PK genes were grouped in 73 clusters distributed unevenly among the 20 soybean chromosomes. Gene IDs and the corresponding subfamily names are indicated. Subfamilies are colour coded and the numbers in the left bar denote chromosomal locations of clusters. (This figure is available in colour at *JXB* online.)

We searched for PK genes that were tandemly arranged at the same chromosomal location. We found that 229 genes (10.57%) were clustered at specific chromosomal locations (Supplementary Table S8 at *JXB* online), suggesting that they may have been generated from tandem duplications. The tandemly arrayed genes were found in 73 clusters distributed unevenly among the 20 soybean chromosomes (Fig. 4), where chromosomes 6, 8, 13, and 18 contained the highest number of genes. The largest cluster was identified on chromosome 6 and contained 14 genes all of which belong to the RLK-Pelle_DLSV subfamily (Fig. 4). Interestingly, the 229 genes were distributed into 24 subfamilies, from which 21 were assigned to the RLK group, indicating that tandem duplication mainly contributed to the expansion of RLK group. Furthermore, we tested whether tandem duplication events had contributed to the expansion of specific subfamilies. Interestingly, we found that tandem duplications accounted for the generation of 58.3% (7/12 genes) of the RLK-Pelle_RLCK-Os subfamily. Similarly, tandem duplications seem to have contributed to the generation of about 52.2% (12/23 genes) and 46.2% (6/13 genes) of the RLK-Pelle_WAK and RLK-Pelle_RKF3 gene subfamily members, respectively (Supplementary Table S9 at *JXB* online). Also, RLK-Pelle_DLSV and RLK-Pelle_LRR-XI-1 had many genes generated through tandem duplications (Supplementary Table S9).

Previous reports have indicated that PKs expanded via tandem duplication tend to function in biotic stress responses (Hanada *et al.*, 2008; Lehti-Shiu *et al.*, 2009). Therefore, we used soybean Gene Ontology (GO) categorization to examine the functional bias of the 229 tandemly arrayed genes. The most abundant groups corresponded to proteins with functions associated with biotic stress responses (44%), development (19%), and abiotic stress responses (11%) (Supplementary Fig. S4 at *JXB* online).

### Subcellular localization of PKs

**Fig. 5.**
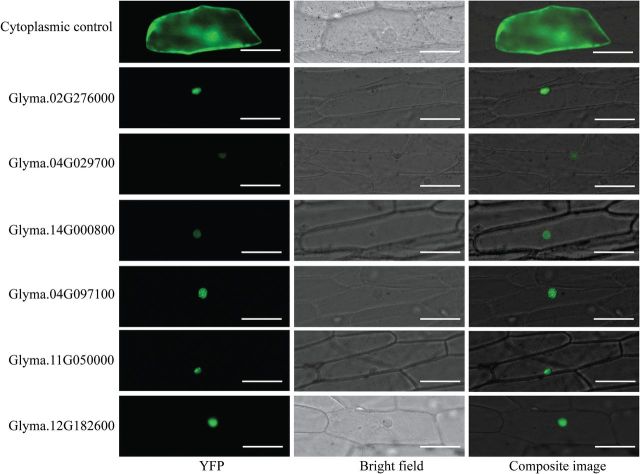
Subcellular localization of soybean PKs in the nucleus of plant cells. The coding sequences of six predicted nucleus-localized soybean PKs were fused to the N terminus of eYFP and expressed in onion epidermal cells via biolistic bombardment. YFP fluorescence was localized exclusively to the nucleus. Bar = 100 μM. (This figure is available in colour at *JXB* online.)

PKs, as one of the main components of signal transduction pathways, are generally involved in perception and transmission of extracellular signals to the nucleus, which results in activation or repression of a specific set of genes involved in a particular cellular process (Antolín-Llovera *et al.*, 2012). With the exception of receptor-like cytoplasmic kinases (RLCKs), which are known to localize to the cytoplasm, RLKs are transmembrane proteins containing extracellular receptor domains and intracellular kinase domains. However, the cellular localizations of the PKs belonging to other groups are largely unknown. Thus, the subcellular localization of these proteins was predicted using WoLF PSORT software (Horton *et al.*, 2007), CELLO (Yu *et al.*, 2006), and NucPred (Brameier *et al.*, 2007). The analysis provided suggestions for extracellular and cytoplasmic as well as nuclear localizations (Supplementary Table S3). Interestingly, several subfamilies included members that were predicted to localize to various cellular compartments, suggesting that gene subfamily members might have experienced subfunctionalization or neofunctionalization. In contrast, members belonging to 11 PK subfamilies were predicted to have the same cellular localization. One striking finding was that 312 proteins were predicted as nucleus-localized kinases (Supplementary Table S3). This number is surprisingly high because, unlike animals, plant systems contain a very limited number of exclusively nucleus-localized PKs (Dahan *et al.*, 2010). Therefore, we tested the subcellular localization of six predicted nucleus-localized PKs *in planta* using onion epidermal cells. The coding sequences of these genes were fused to the N terminus of an enhanced YFP (eYFP) reporter gene and delivered into onion epidermal cells using biolistic bombardment. The YFP signals accumulated exclusively in the nucleus, confirming the predicted subcellular localization of these proteins (Fig. 5).

### Expression patterns of PK families

**Fig. 6.**
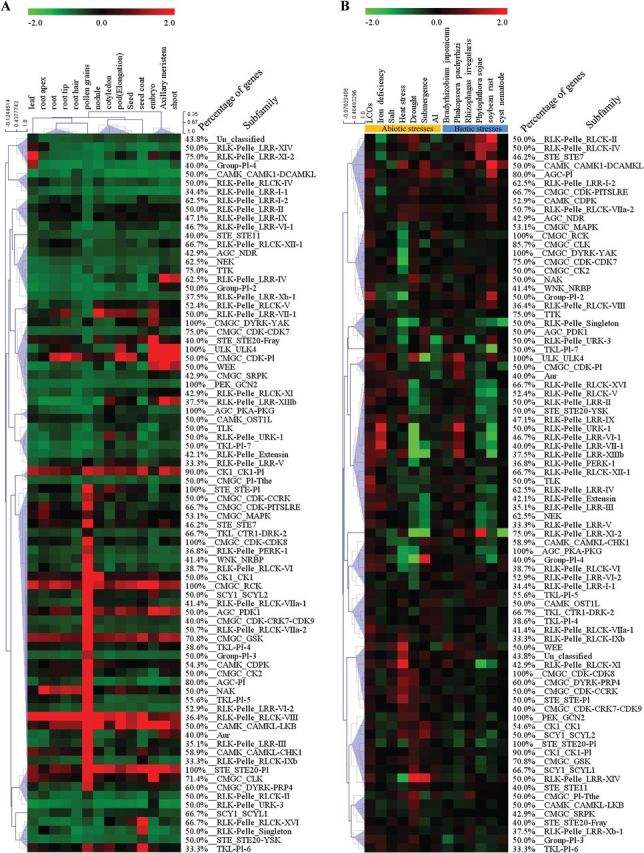
Heat maps of the expression patterns of soybean PK subfamilies in different tissues and under various biotic and abiotic stress conditions. The expression patterns of 82 PK subfamilies in different tissues (A) and under various biotic and abiotic stress conditions (B) are shown. The heat maps were generated using MeV software, v. 4.9. A colour scale corresponding to upregulation and downregulation is shown. (This figure is available in colour at *JXB* online.)

We analysed gene expression profiles of individual PK genes using publicly available Affymetrix microarray datasets from the GPL4592 platform, which includes 53 experiments with 3785 samples. Gene expression data were available only for 817 PK genes that have probes on the Affymetrix GPL4592 platform. The normalized expression values of these genes were examined in 14 organs and tissues comprising roots, root tips, root apex, root hairs, nodules, cotyledons, leaves, shoots, pollen grains, axillary meristem, embryos, seeds, seed coats, and pods (Supplementary Fig. S5 and Supplementary Table S10 at *JXB* online), as well as under various biotic and abiotic stress treatments (Supplementary Fig. S6 and Supplementary Table S11 at *JXB* online). In general, we found that members belonging to the same PK subfamilies showed similar expression patterns. Therefore, we decided to investigate the expression patterns of PK subfamilies rather than individual genes. Because gene expression data for many PKs are not available, we included in our analysis only subfamilies in which gene expression data were available for at least one-third of the subfamily members. As a result, 82 gene subfamilies were included in our analysis out of 122 total subfamilies. The average expression values of these 82 subfamilies were first examined in the 14 organs and tissues indicated above (Fig. 6A). We observed distinct expression patterns of the kinase subfamilies. One pattern was the high expression levels of several subfamilies in almost all tissues, including CK1_CK1-PI, CMGC_RCK, CMGC_GSK, RLK-Pelle_RLCK-VIII, CAMK_CAMKL-LKB, and STE_STE20-PI, suggesting a key role of these subfamilies in fundamental cellular processes. In contrast, certain subfamilies exhibited tissue-specific expression patterns. For example, in root, root tips, and root hairs, RLK-Pelle_RLCK-VIII, NAK, CMGC_CDK-Pl, CAMK_CAMKL-LKB, CMGC_RCK, and CMGC_GSK gene subfamilies exhibited the highest expression levels among all 82 families included in the analysis, suggesting a role for these subfamilies in root development and functions. Similarly, the CMGC_CDK-Pl subfamily showed the highest expression levels in embryos, seeds, and pods, suggesting a role for genes belonging to this subfamily in seed development. One remarkable finding was that many kinase subfamilies exhibited high expression in pollen. Among these subfamilies, RLK-Pelle_LRR-VI-2, RLK-Pelle_RLCK-VIIa-1, TKL-Pl-4, AGC-Pl, RLK-Pelle_RLCK-VI, and CMGC_CDK-CRK7-CDK9 were probably pollen specific because their expression was not detected in the other tissues tested. Similarly, Group-Pl-4, RLK-Pelle_Singleton, and CMGC_DYRK-YAK seemed to be specific to leaf, seed coat, and embryo, respectively, as a high abundance of transcripts belonging to these subfamilies was detected only in these tissues. Notably, the CAMK_CAMK1-DCAMKL, RLK-Pelle_LRR-Xb-1, PEK_GCN2, RLK-Pelle_Extensin, CMGC_P1-The, RLK-Pelle_LRR-I-1, CMGC_CDK-CDK7, STE_STE20-YSK, NEK, STE_STE11, and AGC_NDR subfamilies showed the lowest expression levels across almost all tissues tested.

When we analysed the expression of the 82 kinase subfamilies under various biotic and abiotic stress treatments, these subfamilies showed apparent responses to specific biotic and/or abiotic stress treatments with up- or downregulation (Fig. 6B). For example, the AGC_NDR subfamily was induced by heat stress, drought,and soybean rust. CMGC_CDK-CDK7 showed upregulation in response to drought and salt treatments and downregulation in response to heat stress. The CAMK_CAMK1-DCAMKL subfamily was induced in response to several abiotic/biotic stresses. Similarly, genes of the RLK-Pelle_Extensin subfamily exhibited upregulation in response to lipo-chitooligosaccharides (LCOs) and iron deficiency and downregulation in response to submergence. NEK and RLK-Pelle_LRR-Xb-1 showed upregulation by LCO treatment. PEK_GCN2 and CMGC_Pl-The were highly induced by heat and drought. Certain subfamilies responded specifically to particular stress conditions. For example, TLK, RLK-Pelle_LRR-VII-1, RLK-Pelle_RLCK-XI, and Group-PI-4 were predominantly upregulated in response to LCOs, iron deficiency, heat, and submergence treatments, respectively (Fig. 6B). Similarly, RLK-Pelle_LRR-XI-2 and RLK-Pelle_RLCK-II were specifically upregulated in response to infection by *Phytophthora sojae* and soybean rust, respectively. Consistent with the expected redundant and overlapping functions of PKs, we found that various subfamilies, specifically those belonging to the same groups, responded similarly to different stress conditions, pointing to common signalling pathways controlled by these subfamilies.

### A global gene co-expression network of soybean PK families

**Fig. 7.**
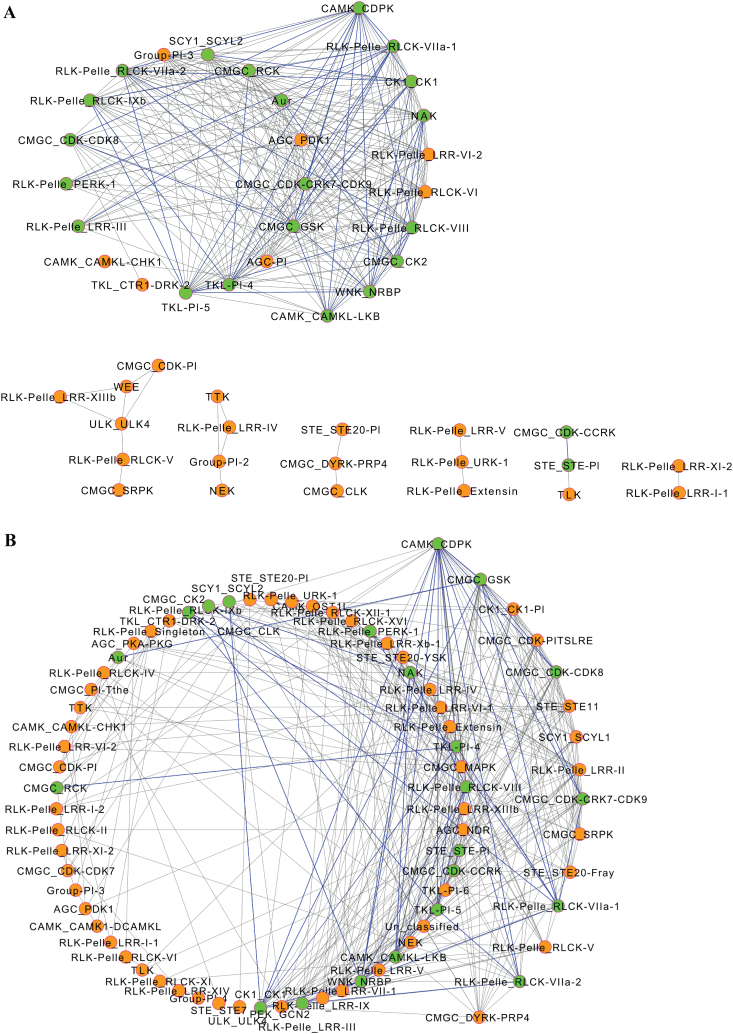
Co-expression networks of soybean PK subfamilies. Two independent co-expression networks were generated using tissue (A) and stress response (B) gene expression data. Nodes indicate subfamilies and edges indicate significant co-expression between subfamilies. Green nodes with blue-coloured edges indicate co-expression events between subfamilies that are common in tissue and stress networks. (This figure is available in colour at *JXB* online.)

The gene expression patterns above pointed to potential co-expression relationships between PK subfamilies. Thus, we constructed the co-expression network of the soybean PK subfamilies using the microarray datasets described above. Construction of the network was performed by determining all pairwise gene expression correlations between PK subfamilies using PCC values and a *P* value of <0.01. Two independent co-expression networks were generated using tissue and stress response gene expression data. The tissue co-expression network contained 48 nodes (kinase subfamilies) and 275 edges (co-expression events) (Fig. 7A) separated into one main and six subnetworks. The frequency distribution of co-expression events for each subfamily revealed both low and broad interconnected relationships. Out of the 27 subfamilies included in the main network, 25 had at least 10 edges and were thus considered hubs. The other six subnetworks contained between two and six subfamilies with the number of edges ranging between one and four. The narrow interconnectivity of these subfamilies implied their association with specific developmental processes.

The stress response co-expression network contained 76 nodes and 364 edges (Fig. 7B). Fifteen PK subfamilies including CAMK_CDPK, CMGC_GSK, CK1_CK1-Pl, CMGC_CDK-PITSLRE, CMGC_CDK-CDK8, STE_STE11, SCY1_SCYL1, CMGC_CDK-CRK7-CDK9, RLK-Pelle_LRR-II, CMGC_SRPK, RLK-Pelle_RLCK-V, RLK-Pelle_RLCK-VIIa-1, RLK-Pelle_RLCK-VIIa-2, and STE_STE20-Fray were identified as having the highest connectivity in the network with at least 15 edges, suggesting their implication in a wide range of signalling pathways regulating soybean response to stress. Finally, when we compared the tissue and stress response co-expression networks we identified 22 subfamilies with 44 co-expression events that were common (Fig. 7), suggesting a role for these subfamilies in coordinating developmental processes and stress signalling.

## Discussion

Our genome-wide bioinformatic analysis identified 2166 putative soybean PKs, which represents 4.67 % of all protein-coding genes in the genome. This proportion is higher than that of *Arabidopsis* (3.7%), maize (3.8%), and rice (4.1%) (Lehti-Shiu and Shiu, 2012; Wei *et al.*, 2014). The size of the soybean PK superfamily is also apparently among the largest in plants. For example, There are 1008 PKs in *Arabidopsis*, 911 members in *M. truncatula*, 1417 members in rice, and 1241 members in maize (Lehti-Shiu and Shiu, 2012; Wei *et al.*, 2014). The large size of the soybean PK superfamily could partly be attributed to the large genome size and two WGD events that occurred 59 and 13 Mya. In addition, high retention of duplicate genes could be another contributing factor. In fact, about 75% of the 46 430 protein-coding genes predicted in soybean were found in multiple copies and are generally arranged in duplicated blocks (Schmutz *et al.*, 2010). Consistent with these data, our analysis revealed that segmental duplication events have played the key role in expanding the soybean PK repertoire and account for the generation of 71.42% of the soybean kinome. Tandem duplications also might have contributed to the expansion of the soybean kinome but to a much less extent, and their role appears to be restricted to specific subfamilies. It should be noted that subfunctionalization of kinase duplicates and insufficient time for pseudogenization or degeneration may also have contributed to the large size of the soybean PK superfamily. In accordance with this suggestion we found that *K*_a_ */K*_s_ values of the syntenically duplicated PKs contributed by the WGD events occurring 59 and 13 Mya were <1, an indication of purifying selection rather than neutral selection for which the *K*_a_ */K*_s_ value is expected to be equal to 1 (Li *et al.*, 1981; Shiu *et al.*, 2004).

The large numbers of soybean PKs relative to other plant species can be explained mainly by the large numbers of members of a few PK groups. The RLK group in soybean is remarkably large, in that it contains 1418 genes. Similarly, over half of the 2500 PKs in *Eucalyptus grandis* are RLK members (Lehti-Shiu and Shiu, 2012). Previous studies have indicated that this group has been subjected to extensive expansion over the course of plant evolution (Shiu *et al.*, 2004; Gao and Xue, 2012; Lehti-Shiu and Shiu, 2012; Wei *et al.*, 2014). The expansion of the RLK/Pelle group appears to be associated with specific families within this group, including DLSV, LRR, and RLCK, which contain far more members in soybean than in other plant species. LRR is the largest family within the RLK-Pelle group in soybean with 479 members, which are grouped into 23 subfamilies. Functional characterization of several members of these gene families in a number of plant species has revealed their implication in a wide range of biological processes including pathogen recognition, organ patterns, steroid signalling, and stem cell control (reviewed by Gish and Clark, 2011).

The dramatic expansion of the LRR subfamilies in soybean is very remarkable and may point to a specific adaptation to sense and respond to variable developmental signals and rapidly evolving pathogen secretions. One obvious exception is the LRR-XII-1 subfamily, which contains 3.8 times more members in rice than in soybean (96 in rice versus 25 in soybean) (Fig. 2). The LRR-XII subfamily is also overrepresented in tomato (Sakamoto *et al.*, 2012), and the expansion of this subfamily in cultivated plants such as tomato and rice could be the result of intense selection for disease-resistant varieties. Members of this subfamily have a well-known role in disease resistance, including EF-Tu RECEPTOR (EFR) and FLAGELLIN-SENSITIVE 2 (FLS2) in *Arabidopsis*, and Xa21 in rice (Shiu *et al.*, 2004; Sakamoto *et al.*, 2012). RLCK is the second largest subfamily of the RLK/Pelle group and contains 267 members belonging to 16 subfamilies.

Unlike most of the RLK/Pelle families, which are membrane-located, RLCKs are localized to the cytoplasm because of the absence of extracellular and transmembrane domains (Jurca *et al.*, 2008). A limited number of RLCKs have been functionally studied, but some well-characterized RLCK genes indicate their implication in disease resistance, abiotic stress response, and hormone signalling (Tang *et al.*, 1996; Shen *et al.*, 2001; Swiderski and Innes, 2001; Muto *et al.*, 2004; Yang *et al.*, 2004). The DLSV subfamily also exhibits considerable differential expansion in soybean and is comprised of 238 members relative to only 89 members in *Arabidopsis* and 146 in rice. Initially, it was suggested that RLK/Pelle duplicated genes are those with defence/resistance-related functions, whereas those implicated in developmental control have been hardly duplicated (Shiu *et al.*, 2004). It is widely known that pathogen recognition receptors are generally expressed at very low levels (Meyers *et al.*, 2002) because high expression of these genes can lead to cell death and dwarf phenotypes (Chern *et al.*, 2005; Kim and Wang, 2010), and hence low expression of RLK/Pelle duplicated genes is anticipated. Nevertheless, recent studies indicated that the RLK/Pelle gene family tends to be enriched in genes that are highly responsive to biotic and abiotic stresses and during various vegetative and reproductive developmental stages (Vij *et al.*, 2008; Lehti-Shiu *et al.*, 2009; Gao and Xue, 2012; Wei *et al.*, 2014). Similarly, our gene expression analysis indicated that many subfamilies of DLSV, LRR, and RLCK are differentially expressed in a number of soybean tissues and organs and in response to different biotic and abiotic stress treatments. Taken together, these data argue against the hypothesis that the majority of RLK/Pelle duplicated genes are implicated in defence/resistance functions but rather suggest that large-scale expansion of this group, including DLSV, LRR, and RLCK families, in soybean may represent a specific adaptation to detect and respond to both developmental signals and environmental stimuli by changing cellular metabolism, gene expression, or growth and development. In support with this suggestion, several RLK-Pelle subfamilies displayed the highest connectivity in both tissue and stress co-expression networks, suggesting a role in synchronizing the interactions between defence signalling and plant growth and developmental pathways.

It has been suggested that lineage-specific expansion of biotic stress-responsive RLKs has been generated through tandem duplications because tandem duplications can initiate quick variations in gene content relative to other types of duplication (Hanada *et al.*, 2008; Lehti-Shiu *et al.*, 2009). In this context, it may be important to mention that all PK genes that are arranged as tandem repeats mainly belong to the RLK group. Thus, tandem duplications seem to have contributed to the expansion of the RLK group in soybean even though these tandemly arranged genes constitute only 16% of the RLK members. In *Arabidopsis*, both tandem and segmental duplications have contributed equally to the large number of RLK members, while in rice only tandem duplications have been assumed to play a major role in RLK expansion (Shiu *et al.*, 2004). When the soybean tandemly repeated PK genes were grouped by associated biological processes using GO categorization, we found that the most abundant group corresponded to defence responses. These data are consistent with previous studies that RLKs with a role in defence tend to be in tandem repeats (Shiu *et al.*, 2004; Lehti-Shiu *et al.*, 2009).

Other non-RLK/Pelle subfamilies appear to be expanded through lineage-specific and species-specific gene duplication to cope with specific environmental stresses. In this regard, we identified 31 subfamilies in which expansion events had been contributed only by the 13-Mya WGD (Supplementary Table S6). Among these subfamilies, the extensive expansion of several TKLs was very apparent. The TKL family in soybean has 115 members compared with 55 and 57 members in rice and *Arabidopsis*, respectively. While none of the soybean TKL genes has been functionally characterized, our gene expression data point to a functional role of these family members in the abiotic stress response, specifically aluminium toxicity and iron deficiency. Similarly, various subfamilies of the well-expanded STE family exhibited more restricted, abiotic stress-specific patterns of expression. Therefore, the extensive expansion of specific PK families in soybean could be the result of strong selection pressure for adaptation to a wide range of environments. Other widely expanded gene subfamilies such as AGC_RSK-2, CAMK_CAMKL-CHK1, and CAMK_CDPK were expressed in at least one tissue type and induced by one or more stress treatments.

The expression patterns of the extensively expanded families suggested that the kinome of soybean has been shaped by its developmental habits and physiological characteristics. Consistent with this hypothesis, we found that almost all of the soybean PK subfamilies were highly upregulated in response to LCOs. LCOs are rhizobial signalling molecules playing a fundamental role in the initiation of nitrogen-fixing symbiosis in legumes. LCOs are perceived by several receptors in host roots and initiate a cascade of signalling events leading to the formation of root nodules (Oldroyd and Downie, 2008). While the pronounced response of soybean PKs to LCOs is in agreement with the well-documented effects of LCOs on plant development and morphogenesis as well as biotic and abiotic stress responses (Wang *et al.*, 2012*a*), it provides the foundation to speculate that PK genes may have been co-opted to regulate a wide range of biological functions including symbiotic interactions. In addition to the RLK/Pelle family, the soybean kinome contains a significant percentage (7.29%) of CAMKs with 70 genes coding for calcium-dependent PKs (CDPK). The expansion of this family may reflect an adaptive evolution that allows soybean plants to perceive different calcium signals that mediate the plant response to environmental stress. On the other hand, several PK subfamilies have been subjected to limited expansion in soybean and remained low in copy number. These subfamilies probably represent ancient families in which new members have not been retained because they did not confer a selective advantage over the ancestral copy. Alternatively, these subfamilies may be involved in more basic and less environment-dependent cellular processes where the driving forces behind continuous expansion and evolution are very limited (Shiu *et al.*, 2004).

Domain organization and intron/exon arrangement have frequently been used as a supporting indication for evolutionary relationships among genes and species. Domain organization analysis revealed that 74 PK genes contained two or three kinase domains. Because many PKs are known to form homo- and heterodimers (Ding *et al.*, 2009), the two- and three- kinase domain structure of soybean PKs may be functionally equivalent to dimerized PKs where cooperative activity of two or three kinase domains may be required for specific substrates. Functional studies of truncated variants of these PKs will provide valuable insights into their mechanism of regulation and substrate specificity. The gene structure of the PK subfamilies clearly elucidated their differential propensity to lose or acquire introns. We found that the proportion of intronless PK genes (12.1%, 262 genes) was much less than that of total intronless genes in the soybean genome (21.7%), suggesting that intron gain has significantly contributed to the structural divergence of the soybean PK superfamily. It seems likely that the structural evolution of soybean PKs is conditioned by similar mechanisms controlling intron gain and loss in both monocot and eudicot species because the patterns of intron number distribution are very similar (Supplementary Fig. S3). We observed that intron/exon position and arrangement patterns were tightly conserved in a number of subfamilies, specifically those whose expansion had been contributed by the WGD occurring 13 Mya, consistent with their close evolutionary relationship. In contrast, other members of subfamilies displayed a high structural diversity but a strong conservation of the kinase domain. This may reflect gene family expansion from old paralogues or multiple ancestral origins of the gene family. Finally, the wide structure differences of the PK supergene family associated with functional divergence may have contributed significantly to the high retention of the duplicate genes in soybean.

In conclusion, our analyses of soybean PKs revealed their wide expansion, and extensive divergence in gene structure, tissue and stress gene expression responses, and subcellular localizations. Functional characterization of subfamily members using high-throughput reverse genetics approaches will facilitate deciphering the multifunctional roles of the soybean kinome in plant development and physiology.

## Supplementary data

Supplementary data are available at *JXB* online.

Supplementary Table S1. Kinase domain annotation of 2166 soybean protein kinases.

Supplementary Table S2. List of 186 atypical soybean kinases.

Supplementary Table S3. Subfamily classification of soybean protein kinases and their related information.

Supplementary Table S4. Conserved domains of soybean protein kinases.

Supplementary Table S5. List of 74 soybean protein kinases containing two or three kinase domains.

Supplementary Table S6. Collinearity events and *K*_a_ */K*_s_ values of soybean protein kinases.

Supplementary Table S7. List of 31 protein kinase subfamilies specific to the 13-Mya WGD.

Supplementary Table S8. List of 229 tandemly arrayed soybean protein kinases.

Supplementary Table S9. Contribution of tandem duplication to the expansion of RLK group.

Supplementary Table S10. Normalized gene expression values of 817 PK genes in different tissues.

Supplementary Table S11. Normalized gene expression values of 817 PK genes under various biotic and abiotic stress conditions.

Supplementary Fig. S1. Phylogenetic classification of soybean protein kinases using the neighbour-joining method.

Supplementary Fig. S2. Phylogenetic classification of soybean protein kinases using the maximum-likelihood method.

Supplementary Fig. S3. Patterns distribution of intron numbers of protein kinase gene family in soybean and other plant species.

Supplementary Fig. S4. GO-term annotation of 229 tandemly arrayed soybean protein kinases.

Supplementary Fig. S5. Heat map of the expression patterns of individual soybean PK genes in different tissues.

Supplementary Fig. S6. Heat map of the expression patterns of individual soybean PK genes under various biotic and abiotic stress conditions.

Supplementary Data

## Abbreviations:

AGC: PKA–PKG–PKC
CAMK: calcium- and calmodulin-regulated kinase
CDPK: calcium-dependent PKs;
CK1: casein kinase 1
CMGC: cyclin-dependent kinases, mitogen-activated protein kinases, glycogen synthase kinase and cyclin-dependent kinases
eYFP: enhanced yellow fluorescent protein
GO: Gene Ontology
HMM: hidden Markov model
LCO: lipo-chitooligosaccharide
LRR: leucine-rich repeat
ML: maximum likelihood
Mya: million years ago
NJ: neighbour joining
PCC: Pearson correlation coefficient
PK: protein kinase
RLCK: receptor-like cytoplasmic kinase
RLK: receptor-like kinase
TK: tyrosine kinase
TKL: tyrosine kinase-like kinase
WGD: whole-genome-wide duplication.

## References

[CIT0001] Antolín-Llovera M Ried MK Binder A Parniske M 2012 Receptor kinase signaling pathways in plant–microbe interactions. Annual Review of Phytopathology 50, 451–473. 10.1146/annurev-phyto-081211-173002 22920561

[CIT0002] Brameier M Krings A MacCallum RM 2007 NucPred—predicting nuclear localization of proteins. Bioinformatics 23, 1159–1160. 17332022 10.1093/bioinformatics/btm066

[CIT0003] Champion A Kreis M Mockaitis K Picaud A Henry Y 2004 Arabidopsis kinome: after the casting. Functional and Integrative Genomics 4, 163–187. 14740254 10.1007/s10142-003-0096-4

[CIT0004] Chern M Fitzgerald HA Canlas PE Navarre DA Ronald PC 2005 Overexpression of a rice NPR1 homolog leads to constitutive activation of defense response and hypersensitivity to light. Molecular Plant–Microbe Interactions 18, 511–520. 15986920 10.1094/MPMI-18-0511

[CIT0005] Colcombet J Hirt H 2008 Arabidopsis MAPKs: a complex signalling network involved in multiple biological processes. Biochemical Journal 413, 217–226. 18570633 10.1042/BJ20080625

[CIT0006] Dahan J Wendehenne D Ranjeva R Pugin A Bourque S 2010 Nuclear protein kinases: still enigmatic components in plant cell signalling. New Phytologist 185, 355–368. 19925553 10.1111/j.1469-8137.2009.03085.x

[CIT0007] Ding X Richter T Chen M 2009 A rice kinase-protein interaction map. Plant Physiology 149, 1478–1492. 19109415 10.1104/pp.108.128298 PMC2649385

[CIT0008] Finn RD Clements J Eddy SR 2011 HMMER web server: interactive sequence similarity searching. Nucleic Acids Research 39, W29–W37. 21593126 10.1093/nar/gkr367 PMC3125773

[CIT0009] Finn RD Mistry J Tate J 2010 The Pfam protein families database. Nucleic Acids Research 38, D211–D222. 19920124 10.1093/nar/gkp985 PMC2808889

[CIT0010] Freeling M 2009 Bias in plant gene content following different sorts of duplication: tandem, whole-genome, segmental, or by transposition. Annual Review of Plant Biology 60, 433–453. 10.1146/annurev.arplant.043008.092122 19575588

[CIT0011] Gao LL Xue HW 2012 Global analysis of expression profiles of rice receptor-like kinase genes. Molecular Plant 5, 143–153. 21765177 10.1093/mp/ssr062

[CIT0012] Gautier L Cope L Bolstad BM Irizarry RA 2004 affy—analysis of Affymetrix GeneChip data at the probe level. Bioinformatics 20, 307–315. 14960456 10.1093/bioinformatics/btg405

[CIT0013] Gish LA Clark SE 2011 The RLK/Pelle family of kinases. The Plant Journal 66, 117–127. 21443627 10.1111/j.1365-313X.2011.04518.x PMC4657737

[CIT0014] Hanada K Zou C Lehti-Shiu MD Shinozaki K Shiu SH 2008 Importance of lineage-specific expansion of plant tandem duplicates in the adaptive response to environmental stimuli. Plant Physiology 148, 993–1003. 18715958 10.1104/pp.108.122457 PMC2556807

[CIT0015] Hanks SK Hunter T 1995 Protein kinases 6. The eukaryotic protein kinase superfamily: kinase (catalytic) domain structure and classification. FASEB Journal 9, 576–596. 7768349

[CIT0016] Hanks SK Quinn AM Hunter T 1988 The protein kinase family: conserved features and deduced phylogeny of the catalytic domains. Science 241, 42–52. 3291115 10.1126/science.3291115

[CIT0017] Hewezi T Howe PJ Maier TR Hussey RS Mitchum MG Davis EL Baum TJ 2010 Arabidopsis spermidine synthase is targeted by an effector protein of the cyst nematode *Heterodera schachtii* . Plant Physiology 152, 968–984. 19965964 10.1104/pp.109.150557 PMC2815906

[CIT0018] Horton P Park KJ Obayashi T Fujita N Harada H Adams-Collier CJ Nakai K 2007 WoLF PSORT: protein localization predictor. Nucleic Acids Research 35, W585–W587. 17517783 10.1093/nar/gkm259 PMC1933216

[CIT0019] Im JH Lee H Kim J Kim HB An CS 2012 Soybean MAPK, GMK1 is dually regulated by phosphatidic acid and hydrogen peroxide and translocated to nucleus during salt stress. Molecules and Cells 34, 271–278. 22886763 10.1007/s10059-012-0092-4 PMC3887844

[CIT0020] Jurca ME Bottka S Feher A 2008 Characterization of a family of Arabidopsis receptor-like cytoplasmic kinases (RLCK class VI). Plant Cell Reports 27, 739–748. 18087702 10.1007/s00299-007-0494-5

[CIT0021] Kim TW Wang ZY 2010 Brassinosteroid signal transduction from receptor kinases to transcription factors. Annual Review of Plant Biology 61, 681–704. 10.1146/annurev.arplant.043008.092057 20192752

[CIT0022] Krogh A Larsson B von Heijne G Sonnhammer ELL 2001 Predicting transmembrane protein topology with a hidden Markov model: application to complete genomes. Journal of Molecular Biology 305, 567–580. 11152613 10.1006/jmbi.2000.4315

[CIT0023] Lan P Li WF Schmidt W 2013 Genome-wide co-expression analysis predicts protein kinases as important regulators of phosphate deficiency-induced root hair remodeling in Arabidopsis. BMC Genomics 14, 210. 23547783 10.1186/1471-2164-14-210 PMC3636113

[CIT0024] Lehti-Shiu MD Shiu SH 2012 Diversity, classification and function of the plant protein kinase superfamily. Philosophical Transactions of the Royal Society B—Biological Sciences 367, 2619–2639. 10.1098/rstb.2012.0003 PMC3415837 22889912

[CIT0025] Lehti-Shiu MD Zou C Hanada K Shiu SH 2009 Evolutionary history and stress regulation of plant receptor-like kinase/pelle genes. Plant Physiology 150, 12–26. 19321712 10.1104/pp.108.134353 PMC2675737

[CIT0026] Li WH Gojobori T Nei M 1981 Pseudogenes as a paradigm of neutral evolution. Nature 292, 237–239. 7254315 10.1038/292237a0

[CIT0027] Lin WD Liao YY Yang TJ Pan CY Buckhout TJ Schmidt W 2011 Coexpression-based clustering of Arabidopsis root genes predicts functional modules in early phosphate deficiency signaling. Plant Physiology 155, 1383–1402. 21248074 10.1104/pp.110.166520 PMC3046593

[CIT0028] Liu JZ Braun E Qiu WL Shi YF Marcelino-Guimaraes FC Navarre D Hill JH Whitham SA 2014 Positive and negative roles for soybean MPK6 in regulating defense responses. Molecular Plant–Microbe Interactions 27, 824–834. 24762222 10.1094/MPMI-11-13-0350-R

[CIT0029] Liu JZ Horstman HD Braun E 2011 Soybean homologs of MPK4 negatively regulate defense responses and positively regulate growth and development. Plant Physiology 157, 1363–1378. 21878550 10.1104/pp.111.185686 PMC3252160

[CIT0030] Maere S De Bodt S Raes J Casneuf T Van Montagu M Kuiper M Van de Peer Y 2005 Modeling gene and genome duplications in eukaryotes. Proceedings of the National Academy of Sciences, USA 102, 5454–5459. 10.1073/pnas.0501102102 PMC556253 15800040

[CIT0031] Manning G Plowman GD Hunter T Sudarsanam S 2002 Evolution of protein kinase signaling from yeast to man. Trends in Biochemical Sciences 27, 514–520. 12368087 10.1016/s0968-0004(02)02179-5

[CIT0032] Meyers BC Morgante M Michelmore RW 2002 TIR-X and TIR-NBS proteins: two new families related to disease resistance TIR-NBS-LRR proteins encoded in Arabidopsis and other plant genomes. The Plant Journal 32, 77–92. 12366802 10.1046/j.1365-313x.2002.01404.x

[CIT0033] Morillo SA Tax FE 2006 Functional analysis of receptor-like kinases in monocots and dicots. Current Opinion in Plant Biology 9, 460–469. 16877029 10.1016/j.pbi.2006.07.009

[CIT0034] Muto H Yabe N Asami T Hasunuma K Yamamoto KT 2004 Overexpression of constitutive differential growth 1 gene, which encodes a RLCKVII-subfamily protein kinase, causes abnormal differential and elongation growth after organ differentiation in Arabidopsis. Plant Physiology 136, 3124–3133. 15466232 10.1104/pp.104.046805 PMC523373

[CIT0035] Niedner RH Buzko OV Haste NM Taylor A Gribskov M Taylor SS 2006 Protein kinase resource: an integrated environment for phosphorylation research. Proteins—Structure Function and Bioinformatics 63, 78–86. 10.1002/prot.20825 16435372

[CIT0036] Oldroyd GED Downie JM 2008 Coordinating nodule morphogenesis with rhizobial infection in legumes. Annual Review of Plant Biology 59, 519–546. 10.1146/annurev.arplant.59.032607.092839 18444906

[CIT0037] Petersen TN Brunak S von Heijne G Nielsen H 2011 SignalP 4.0: discriminating signal peptides from transmembrane regions. Nature Methods 8, 785–786. 21959131 10.1038/nmeth.1701

[CIT0038] Rodriguez MC Petersen M Mundy J 2010 Mitogen-activated protein kinase signaling in plants. Annual Review of Plant Biology 61, 621–649. 10.1146/annurev-arplant-042809-112252 20441529

[CIT0039] Saito R Smoot ME Ono K Ruscheinski J Wang PL Lotia S Pico AR Bader GD Ideker T 2012 A travel guide to Cytoscape plugins. Nature Methods 9, 1069–1076. 23132118 10.1038/nmeth.2212 PMC3649846

[CIT0040] Sakamoto T Deguchi M Brustolini OJB Santos AA Silva FF Fontes EPB 2012 The tomato RLK superfamily: phylogeny and functional predictions about the role of the LRRII-RLK subfamily in antiviral defense. BMC Plant Biology 12, 229. 23198823 10.1186/1471-2229-12-229 PMC3552996

[CIT0041] Schmid M Davison TS Henz SR Pape UJ Demar M Vingron M Scholkopf B Weigel D Lohmann JU 2005 A gene expression map of *Arabidopsis thaliana* development. Nature Genetics 37, 501–506. 15806101 10.1038/ng1543

[CIT0042] Schmutz J Cannon SB Schlueter J 2010 Genome sequence of the palaeopolyploid soybean. Nature 463, 178–183. 20075913 10.1038/nature08670

[CIT0043] Shen W Gomez-Cadenas A Routly EL Ho THD Simmonds JA Gulick PJ 2001 The salt stress-inducible protein kinase gene, Esi47, from the salt-tolerant wheatgrass *Lophopyrum elongatum* is involved in plant hormone signaling. Plant Physiology 125, 1429–1441. 11244122 10.1104/pp.125.3.1429 PMC65621

[CIT0044] Shiu SH Bleecker AB 2003 Expansion of the receptor-like kinase/Pelle gene family and receptor-like proteins in Arabidopsis. Plant Physiology 132, 530–543. 12805585 10.1104/pp.103.021964 PMC166995

[CIT0045] Shiu SH Karlowski WM Pan RS Tzeng YH Mayer KFX Li WH 2004 Comparative analysis of the receptor-like kinase family in Arabidopsis and rice. Plant Cell 16, 1220–1234. 15105442 10.1105/tpc.020834 PMC423211

[CIT0046] Srour A Afzal AJ Blahut-Beatty L 2012 The receptor like kinase at Rhg1-a/Rfs2 caused pleiotropic resistance to sudden death syndrome and soybean cyst nematode as a transgene by altering signaling responses. BMC Genomics 13, 368. 22857610 10.1186/1471-2164-13-368 PMC3439264

[CIT0047] Sun XL Yu QY Tang LL Ji W Bai X Cai H Liu XF Ding XD Zhu YM 2013 GsSRK, a G-type lectin S-receptor-like serine/threonine protein kinase, is a positive regulator of plant tolerance to salt stress. Journal of Plant Physiology 170, 505–515. 23276523 10.1016/j.jplph.2012.11.017

[CIT0048] Swiderski MR Innes RW 2001 The Arabidopsis PBS1 resistance gene encodes a member of a novel protein kinase subfamily. The Plant Journal 26, 101–112. 11359614 10.1046/j.1365-313x.2001.01014.x

[CIT0049] Tang XY Frederick RD Zhou JM Halterman DA Jia YL Martin GB 1996 Initiation of plant disease resistance by physical interaction of AvrPto and Pto kinase. Science 274, 2060–2063. 8953033 10.1126/science.274.5295.2060

[CIT0050] Tena G Boudsocq M Sheen J 2011 Protein kinase signaling networks in plant innate immunity. Current Opinion in Plant Biology 14, 519–529. 21704551 10.1016/j.pbi.2011.05.006 PMC3191242

[CIT0051] Vij S Giri J Dansana PK Kapoor S Tyagi AK 2008 The receptor-like cytoplasmic kinase (OsRLCK) gene family in rice: organization, phylogenetic relationship, and expression during development and stress. Molecular Plant 1, 732–750. 19825577 10.1093/mp/ssn047

[CIT0052] Wang M Vannozzi A Wang G Liang YH Tornielli GB Zenoni S Cavallini E Pezzotti M Cheng ZM 2014 Genome and transcriptome analysis of the grapevine (*Vitis vinifera* L.) WRKY gene family. Horticulture Research 1, 16. 10.1038/hortres.2014.16 PMC4596322 26504535

[CIT0053] Wang N Khan W Smith DL 2012a Changes in soybean global gene expression after application of lipo-chitooligosaccharide from *Bradyrhizobium japonicum* under sub-optimal temperature. PLoS One 7, e31571. 22348109 10.1371/journal.pone.0031571 PMC3278468

[CIT0054] Wang S Yin YB Ma Q Tang XJ Hao DY Xu Y 2012b Genome-scale identification of cell-wall related genes in Arabidopsis based on co-expression network analysis. BMC Plant Biology 12, 138. 22877077 10.1186/1471-2229-12-138 PMC3463447

[CIT0055] Wang YP Tang HB DeBarry JD 2012c MCScanX: a toolkit for detection and evolutionary analysis of gene synteny and collinearity. Nucleic Acids Research 40, e49. 22217600 10.1093/nar/gkr1293 PMC3326336

[CIT0056] Wei KF Wang YM Xie DX 2014 Identification and expression profile analysis of the protein kinase gene superfamily in maize development. Molecular Breeding 33, 155–172.

[CIT0057] Yang L Wu KC Gao P Liu XJ Li GP Wu ZJ 2014 GsLRPK, a novel cold-activated leucine-rich repeat receptor-like protein kinase from *Glycine soja*, is a positive regulator to cold stress tolerance. Plant Science 215, 19–28. 24388511 10.1016/j.plantsci.2013.10.009

[CIT0058] Yang TB Chaudhuri S Yang LH Chen YP Poovaiah BW 2004 Calcium/calmodulin up-regulates a cytoplasmic receptor-like kinase in plants. Journal of Biological Chemistry 279, 42552–42559. 15292241 10.1074/jbc.M402830200

[CIT0059] Yu CS Chen YC Lu CH Hwang JK 2006 Prediction of protein subcellular localization. Proteins—Structure Function and Bioinformatics 64, 643–651. 10.1002/prot.21018 16752418

