# Supplementary material for: Soybean kinome: functional classification and gene expression patterns
Source: J Exp Bot. 2015 Jan 22;66(7):1919–34. doi: 10.1093/jxb/eru537 (PMC4378628; doi:10.1093/jxb/eru537)
Supplement: Supplementary Data [file supp_66_7_1919__index.html]

Soybean kinome: functional classification and gene expression patterns — Soybean kinome: functional classification and gene expression patterns — Supplementary Data 

# Soybean kinome: functional classification and gene expression patterns

## Supplementary Data

Data files

**Files in this Data Supplement:**

- Supplementary Data - Supplementary Data
- Supplementary Data - Supplementary Data
