# Supplementary material for: Soybean kinome: functional classification and gene expression patterns
Source: J Exp Bot. 2015 Jan 22;66(7):1919–34. doi: 10.1093/jxb/eru537 (PMC4378628; doi:10.1093/jxb/eru537)
Supplement: Supplementary Data [file supp_eru537_jexbot136416_file001.pdf]

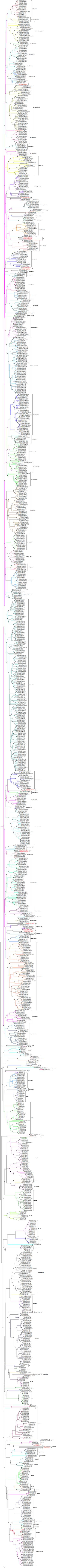

Supplemental Figure 1: Phylogenetic classification of soybean protein kinases using neighbor-joining method. Genes showing different classifications using maximum-likelihood method are highlighted in red.

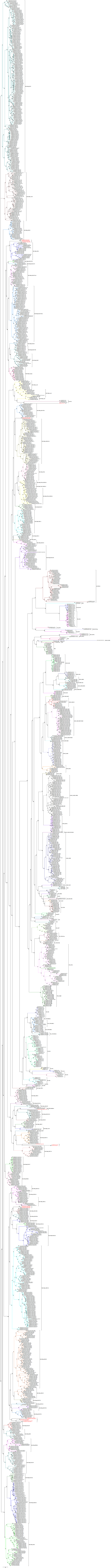

Supplemental Figure 2: Phylogenetic classification of soybean protein kinases using maximum-likelihood method. Genes showing different classification using neighbor-joining method are highlighted in red.

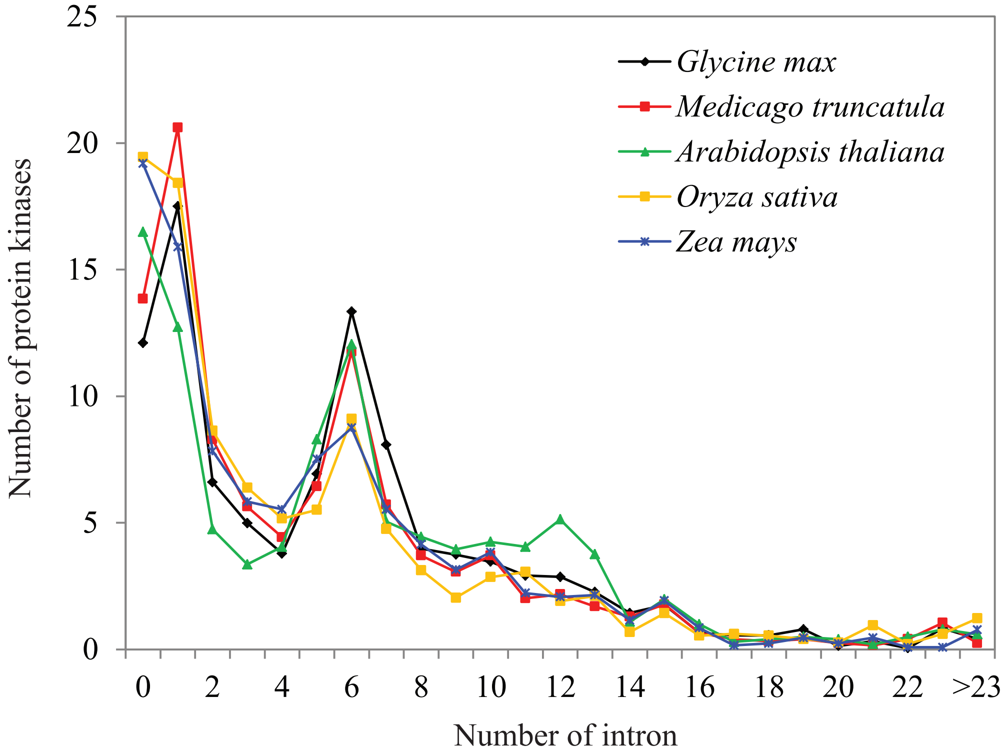

Supplemental Figure 3: Patterns distribution of intron numbers of protein kinase gene family in soybean and other plant species.

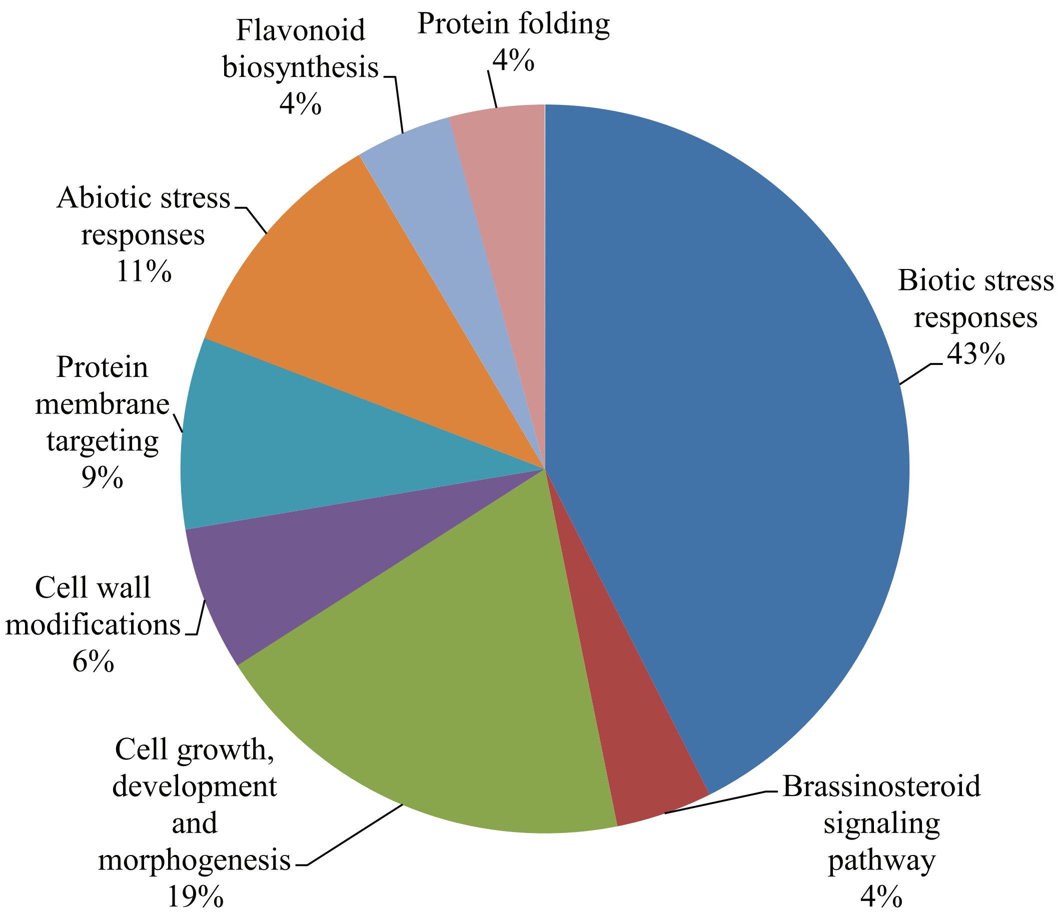

Supplemental Figure 4: GO-term annotation of 229 tandemly arrayed soybean protein kinases. GO terms were generated using BLAST2GO program with filter value of 3 and default paramaters.

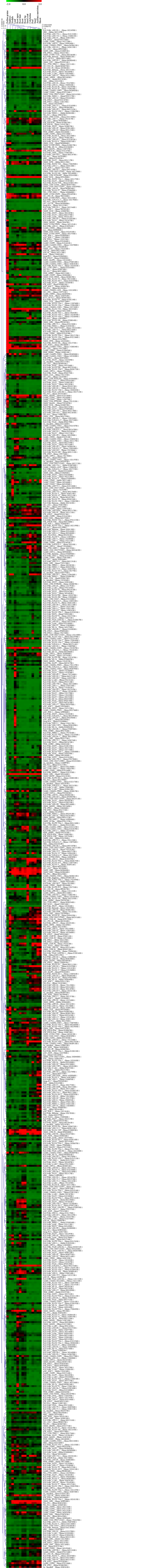

Supplemental Figure 5: Heat map of the expression patterns of individual soybean PK genes in different tissues. The expression patterns of 817 PK genes in different tissues are shown. The heat maps were generated using MeV (Multiple Experiment Viewer) software, version 4.9. Red and green correspond to up-regulation and down-regulation, respectively. Normalized gene expression values are provided in Supplemental Table 10.

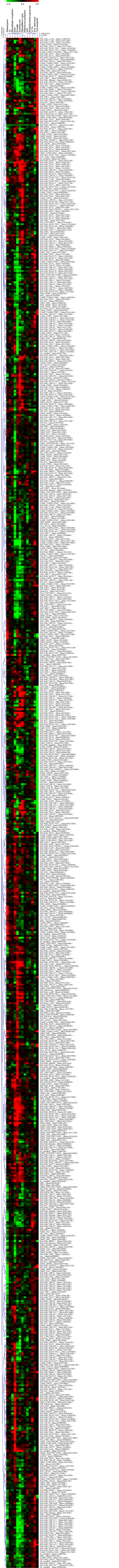

-2.0
0.0
2.0
Submergence
Al
Rhizophagus irregularis
Heat
Drought
Soybean rust
Phytophthora sojae
Phakopsora pachyrhizi
Salt
Bradyrhizobium japonicum
LCOs
Iron_deficiency
Cyst nematode
-0.05591667
0.47204167
1.0
-0.046275973
0.476862
1.0
RLK-Pelle_L-LEC__Glyma.11G089100.1
RLK-Pelle_L-LEC__Glyma.01G155600.1
RLK-Pelle_L-LEC__Glyma.17G218500.1
CAMK_CDPK__Glyma.02G182900.1
RLK-Pelle_LRR-I-2__Glyma.18G275700.1
RLK-Pelle_RLCK-VIIa-2__Glyma.13G110700.1
RLK-Pelle_RLCK-VIIa-2__Glyma.17G048900.1
CAMK_CAMK1-DCAMKL__Glyma.15G222300.1
RLK-Pelle_DLSV__Glyma.13G248300.1
RLK-Pelle_SD-2b__Glyma.04G042400.1
RLK-Pelle_CrRLK1L-1__Glyma.18G272500.1
CAMK_CAMKL-CHK1__Glyma.09G098000.1
RLK-Pelle_LRR-III__Glyma.20G120700.1
CAMK_CAMKL-CHK1__Glyma.15G203700.1
RLK-Pelle_DLSV__Glyma.11G204700.1
CAMK_CAMKL-CHK1__Glyma.14G203000.1
RLK-Pelle_LRR-XIV__Glyma.14G074700.1
CMGC_GSK__Glyma.03G229200.1
RLK-Pelle_LRR-III__Glyma.06G042500.1
CAMK_CAMKL-CHK1__Glyma.02G235100.1
RLK-Pelle_RLCK-VI__Glyma.02G246000.1
AGC_RSK-2__Glyma.19G060500.1
RLK-Pelle_Extensin__Glyma.09G063200.1
RLK-Pelle_LRR-XI-1__Glyma.13G174900.1
RLK-Pelle_DLSV__Glyma.13G284100.1
Group-Pl-4__Glyma.02G100000.1
CAMK_CAMKL-CHK1__Glyma.02G202900.1
RLK-Pelle_PERK-2__Glyma.17G246300.1
RLK-Pelle_RLCK-VIIa-2__Glyma.12G062900.1
RLK-Pelle_LRR-I-2__Glyma.12G137100.1
CAMK_CAMKL-CHK1__Glyma.08G218400.1
STE_STE11__Glyma.04G213000.1
RLK-Pelle_LRR-III__Glyma.14G206000.1
RLK-Pelle_LRR-XI-1__Glyma.18G199000.1
Group-Pl-3__Glyma.16G017300.1
CAMK_CDPK__Glyma.03G251400.1
RLK-Pelle_DLSV__Glyma.01G240900.1
RLK-Pelle_DLSV__Glyma.10G253100.1
CAMK_OST1L__Glyma.08G133600.1
RLK-Pelle_RLCK-VI__Glyma.17G062300.1
RLK-Pelle_RLCK-VI__Glyma.19G152100.1
CMGC_CDK-CRK7-CDK9__Glyma.13G215200.1
CMGC_DYRK-PRP4__Glyma.09G169500.1
RLK-Pelle_DLSV__Glyma.14G026300.1
RLK-Pelle_RLCK-XII-1__Glyma.13G341100.1
STE_STE11__Glyma.05G080900.1
RLK-Pelle_LRR-VII-1__Glyma.01G133300.1
STE_STE11__Glyma.13G084100.1
RLK-Pelle_RLCK-V__Glyma.16G032700.1
AGC_RSK-2__Glyma.12G056000.1
STE_STE11__Glyma.20G142900.1
CAMK_CDPK__Glyma.03G138000.1
CMGC_MAPK__Glyma.U021800.1
CAMK_CDPK__Glyma.08G316500.1
RLK-Pelle_LRK10L-2__Glyma.09G182200.1
RLK-Pelle_RLCK-VIII__Glyma.10G009100.1
AGC_MAST__Glyma.07G103400.1
RLK-Pelle_DLSV__Glyma.13G266100.1
RLK-Pelle_DLSV__Glyma.20G140100.1
RLK-Pelle_SD-2b__Glyma.06G068100.1

Supplemental Figure 6: Heat map of the expression patterns of individual soybean PK genes under various biotic and abiotic stress conditions. The expression patterns of 817 PK genes under various biotic and abiotic stress conditions are shown. The heat maps were generated using MeV (Multiple Experiment Viewer) software, version 4.9. Red and green correspond to up-regulation and down-regulation, respectively. Normalized gene expression values are provided in Supplemental Table 11.
